# Supplementary material for: Assessing geographic controls of hair isotopic variability in human populations: A case-study in Canada
Source: PLoS One. 2020 Aug 10;15(8):e0237105. doi: 10.1371/journal.pone.0237105 (PMC7416927; doi:10.1371/journal.pone.0237105)
Supplement: S1 Table — Note: samples were collected across several years and some dietary questions were only added post year 1 of the collection efforts. (DOCX) [file pone.0237105.s004.docx]

**S1 Table. Demographics and dietary questions answered by the volunteers to the collection scientist.** Note: samples were collected across several years and some dietary questions were only added post year 1 of the collection efforts.

| **Questions** |
| --- |
| Sex: MALE or FEMALE  Age: 18-29; 30-39; 40-49; 50-59; 60-69; 70+  Source of drinking water:  - groundwater (deep or shallow well; how deep?)  - surface water (name of lake or river) |
| Are you a vegetarian or a vegan? If yes, what is your source of protein? |
| Are you a smoker?  If yes: how long have you smoked?  If no: did you ever smoke? If yes, how long ago did you quit? |
| Do you consume bottled water? If yes, how often and in what quantity? |
| How often, and in what quantity, do you consume the following beverages: bottled water, wine, beer, soft drinks, milk, and fruit juice? |
| What types of other beverages do you consume, how often, and in what quantity? |
| Is your hair dyed? YES or NO |
| How often do you consume seafood and/or fish? Please specify types consumed. |
| Have you travelled outside your local area for more than one day in the last year? If yes, please fill in the following details. |
