# Supplementary material for: Assessing geographic controls of hair isotopic variability in human populations: A case-study in Canada
Source: PLoS One. 2020 Aug 10;15(8):e0237105. doi: 10.1371/journal.pone.0237105 (PMC7416927; doi:10.1371/journal.pone.0237105)
Supplement: S2 Table — p-value>0.05 indicates the distribution is not significantly different from normality. (DOCX) [file pone.0237105.s005.docx]

**S2 Table.** **p-values from Shapiro tests assessing the normality of δ^13^C_hair_, δ^15^N_hair_ and δ^34^S_hair_ distribution.** p-value>0.05 indicates the distribution is not significantly different from normality.

| **Isotope** | **p-value** |
| --- | --- |
| δ^13^C | 0.42 |
| δ^15^N | 0.07 |
| δ^34^S | 0.28 |
