# Supplementary material for: Assessing geographic controls of hair isotopic variability in human populations: A case-study in Canada
Source: PLoS One. 2020 Aug 10;15(8):e0237105. doi: 10.1371/journal.pone.0237105 (PMC7416927; doi:10.1371/journal.pone.0237105)
Supplement: S3 Table — p-values less than 0.05 are highlighted in grey. Values in italics represent provinces with unequal variance (Levene’s test). (DOCX) [file pone.0237105.s006.docx]

**S3 Table.** **p-values from t-tests comparing hair δ^15^N_hair_ values from different provinces.** p-values less than 0.05 are highlighted in grey. Values in italics represent provinces with unequal variance (Levene’s test).

| **Province** | **AB** | **SK** | **MB** | **ON** | **QC** | **NB** | **NS** | **NL** |
| --- | --- | --- | --- | --- | --- | --- | --- | --- |
| **British Columbia (BC)** | 0.53 | *0.43* | 0.25 | 0.13 | 0.0076 | 0.29 | *1.7E-07* | 0.043 |
| **Alberta (AB)** |  | *0.70* | 0.13 | 0.43 | 0.069 | 0.17 | *2.7E-07* | 0.027 |
| **Saskatchewan (SK)** |  |  | *0.14* | *0.88* | *0.44* | 0.18 | *3.0E-04* | *0.026* |
| **Manitoba (MB)** |  |  |  | 0.030 | 0.0014 | 0.97 | 0.0028 | 0.23 |
| **Ontario (ON)** |  |  |  |  | 0.34 | 0.051 | *9.6E-09* | 0.0083 |
| **Quebec (QC)** |  |  |  |  |  | 0.0041 | 7.7E-12 | 4.6E-04 |
| **New Brunswick (NB)** |  |  |  |  |  |  | *0.019* | 0.29 |
| **Nova Scotia (NS)** |  |  |  |  |  |  |  | 0.43 |
