# Supplementary material for: Assessing geographic controls of hair isotopic variability in human populations: A case-study in Canada
Source: PLoS One. 2020 Aug 10;15(8):e0237105. doi: 10.1371/journal.pone.0237105 (PMC7416927; doi:10.1371/journal.pone.0237105)
Supplement: S4 Table — p-values less than 0.05 are highlighted in grey. Values in italics represent provinces with unequal variance (Levene’s test). (DOCX) [file pone.0237105.s007.docx]

**S4 Table** **p-values from t-tests comparing δ^13^C_hair_ values from different provinces.** p-values less than 0.05 are highlighted in grey. Values in italics represent provinces with unequal variance (Levene’s test).

| **Province** | **AB** | **SK** | **MB** | **ON** | **QC** | **NB** | **NS** | **NL** |
| --- | --- | --- | --- | --- | --- | --- | --- | --- |
| **British Columbia (BC)** | 0.014 | 0.00053 | 1.6E-06 | 1.0E-13 | 1.0E-13 | *1.5E-06* | *1.1E-11* | 1.7E-11 |
| **Alberta (AB)** |  | 0.16 | 0.0062 | 1.0E-13 | 3.9E-13 | 2.1E-06 | *7.1E-08* | 1.4E-07 |
| **Saskatchewan (SK)** |  |  | *0.20* | 1.6E-10 | 1.4E-05 | *0.0027* | *5.2E-05* | 1.2E-05 |
| **Manitoba (MB)** |  |  |  | 7.5E-07 | 0.0045 | 0.041 | 0.010 | 0.0037 |
| **Ontario (ON)** |  |  |  |  | 0.0044 | 0.046 | 0.031 | 0.55 |
| **Quebec (QC)** |  |  |  |  |  | 1.0 | 0.81 | 0.26 |
| **New Brunswick (NB)** |  |  |  |  |  |  | 0.87 | 0.39 |
| **Nova Scotia (NS)** |  |  |  |  |  |  |  | *0.28* |
