# Supplementary material for: Assessing geographic controls of hair isotopic variability in human populations: A case-study in Canada
Source: PLoS One. 2020 Aug 10;15(8):e0237105. doi: 10.1371/journal.pone.0237105 (PMC7416927; doi:10.1371/journal.pone.0237105)
Supplement: S5 Table — p-values less than 0.05 are highlighted in grey. Values in italics represent provinces with unequal variance (Levene’s test). (DOCX) [file pone.0237105.s008.docx]

**S5 Table.** **p-values from t-tests comparing hair δ^34^S_hair_ values from different provinces.** p-values less than 0.05 are highlighted in grey. Values in italics represent provinces with unequal variance (Levene’s test).

|  | **AB** | **SK** | **MB** | **ON** | **QC** | **NB** | **NS** | **PE** | **NL** |
| --- | --- | --- | --- | --- | --- | --- | --- | --- | --- |
| **British Columbia (BC)** | 0.052 | 1e-12 | 1e-13 | 0.0055 | 2e-7 | 3e-11 | 1e-13 | 0.0036 | 2e-8 |
| **Alberta (AB)** |  | 4e-8 | 1e-13 | 1e-4 | 1e-10 | 6e-13 | 1e-13 | 5e-4 | 2e-10 |
| **Saskatchewan (SK)** |  |  | 0.015 | 5e-12 | 1e-13 | 1e-13 | 1e-13 | 6e-7 | 1e-13 |
| **Manitoba (MB)** |  |  |  | 1e-13 | 1e-13 | 1e-13 | 1e-13 | 4e-8 | 1e-13 |
| **Ontario (ON)** |  |  |  |  | 0.13 | 7e-4 | 1e-4 | 0.14 | 0.0028 |
| **Quebec (QC)** |  |  |  |  |  | 0.0020 | 0.0011 | 0.13 | 0.0024 |
| **New Brunswick (NB)** |  |  |  |  |  |  | 0.83 | 0.92 | 0.68 |
| **Nova Scotia (NS)** |  |  |  |  |  |  |  | 0.85 | 0.55 |
| **Prince Edward Island (PE)** |  |  |  |  |  |  |  |  | 0.86 |
